# Amsacrine-based induction therapy in AML patients with cardiac comorbidities: a retrospective single-center analysis

**DOI:** 10.1007/s00277-023-05111-x

**Published:** 2023-02-07

**Authors:** David Kuron, Alexander Pohlmann, Linus Angenendt, Torsten Kessler, Rolf Mesters, Wolfgang E. Berdel, Matthias Stelljes, Georg Lenz, Christoph Schliemann, Jan-Henrik Mikesch

**Affiliations:** 1 Department of Medicine A, University Hospital Münster, 48149 Münster, Germany; 2 Current Affiliation: Department of Medicine II, University Hospital Schleswig-Holstein, Arnold-Heller-Str. 3, 24105 Kiel, Germany

**Keywords:** Acute myeloid leukemia, AML, Cardiac comorbidities, Induction therapy, Amsacrine

## Abstract

Intensive chemotherapy is the backbone of induction treatment in patients with acute myeloid leukemia (AML). However, AML patients with concomitant cardiac disease may not be eligible for anthracycline-based therapies. In a small cohort of patients, we have previously shown that anthracycline-free, amsacrine-based chemotherapy TAA (thioguanine, cytarabine, amsacrine) may be as effective as cytarabine/daunorubicin for induction therapy in these patients. In this systematic retrospective single-center analysis, we documented the outcome of 31 patients with significant cardiac comorbidities including coronary heart disease or cardiomyopathy receiving TAA as induction chemotherapy. Median (range) ejection fraction (EF) was 48% (30–67%) in this cohort. Patients with EF below 30% were considered unfit for intensive induction therapy. Event-free survival (EFS), overall survival (OS), and relapse-free survival (RFS) were 1.61, 5.46, and 13.6 months respectively. Poor outcome was primarily related to a high early mortality rate within the first 30 days of therapy, mainly caused by infectious complications. TAA cannot be recommended as a substitute of standard induction for AML patients with significant concomitant cardiac disease. In the era of novel agents, alternative strategies (e.g., hypomethylating agents plus venetoclax) should be considered when anthracycline-based regimens are not suitable.

## Introduction

Acute myeloid leukemia (AML) is a disease of the elderly population with a median age of 72 years at diagnosis [1, 2]. Outcome in older patients is poor compared to younger patients, mainly due to higher early mortality and lower remission rates [3]. In addition to adverse genetic and other biological disease characteristics with impact on prognosis in elderly AML, advanced age often entails a higher likelihood for comorbidities that may preclude standard induction chemotherapy [4]. Anthracycline-based chemotherapy is the backbone of induction therapy in AML with the intention to achieve first complete remission before subsequent consolidation treatment with either conventional chemotherapy or hematopoietic stem cell transplantation (HSCT). Due to the high prevalence of concomitant cardiac disease in elderly patients, it can be challenging for clinicians to identify an appropriate induction regimen [5, 6].

Several recommendations have been made for the treatment of AML patients with significant cardiac comorbidities. As long as the ejection fraction (EF) has not fallen below 45%, patients may be treated with standard induction therapy [7]. The risk of clinical heart failure can be decreased by reducing the anthracycline dose or prolonging the anthracycline infusion time without significantly compromising disease control [8, 9]. The use of cardioprotective drugs and optimized supportive therapy may further reduce the risk of complications during intensive induction therapy [10, 11]. However, patients with coronary heart disease or patients after myocardial infarction do not necessarily suffer from reduced EF and even after optimization of cardiac therapy, they remain at high risk for complications [8, 9].

Recently, various novel approaches have emerged based on the lower-intensity treatment backbones azacitidine and decitabine (hypomethylating agents (HMA)) or low-dose cytarabine (LDAC). These include the B-cell lymphoma 2 (BCL2) inhibitor venetoclax, the hedgehog inhibitor glasdegib, as well as FMS-like tyrosine kinase 3 (FLT3)- and isocitrate dehydrogenase (IDH) 1/2-inhibitors [12]. Some of these regimens have shown impressive response rates with comparably moderate toxicity [13–22]. Especially in patients with significant comorbidities, these regimens provide attractive alternatives to standard induction chemotherapies.

In the past, intensive anthracycline-free chemotherapy regimens have been proposed for patients who are considered fit for intensive therapy in principle but show significant cardiac comorbidities [7, 23, 24]. In this context, others and we have reported that replacement of anthracyclines by amsacrine may provide an alternative intensive induction regimen with less cardiac toxicity and without compromising complete remission rates in AML [25–31]. Thus, amsacrine-based induction has been repeatedly used in the past decades in AML patients with cardiac comorbidities but otherwise preserved performance status.

In this retrospective single-center analysis, we re-evaluated outcomes of patients receiving the TAA regimen as induction chemotherapy in a comparatively large cohort of intensively treated AML patients with cardiac comorbidities between 2009 and 2020.

## Data and statistical analysis

Patients with newly diagnosed de novo, secondary, or therapy-related AML (other than acute promyelocytic leukemia) were included. Patients with cardiac comorbidities, including cardiomyopathy, cardiovascular disease, or heart failure, who were not eligible for treatment with cardiotoxic anthracyclines were considered for treatment with amsacrine-based induction chemotherapy TAA (200 mg/m^2^ thioguanine on days 3–9, 210 mg/m^2^ amsacrine on days 3–5 and 200 mg/m^2^ cytarabine over 24 h on days 1–8). Decision for induction therapy was made in the hematological tumor board of our center.

Remission status was defined according to European LeukemiaNet (ELN) criteria [32]. Patients achieving complete remission (CR) upon induction therapy received consolidation treatment with either 2 or 3 courses of intermediate/high-dose cytarabine (IDAC/HDAC) or allogeneic hematopoietic stem cell transplantation (HSCT). IDAC was dosed at 1 g/m^2^ (every 12 h days 1, 3, and 5) except in one patient who received doses of 3 g/m^2^ (HDAC).

Cardiac function of each patient was assessed via transthoracic echocardiography before start of therapy. If only qualitative results were available, numeric equivalents were chosen according to the guidelines of the British Society of Echocardiography [33].

Survival times were plotted using Kaplan–Meier curves. Relapse-free survival (RFS) was calculated from the time of CR/CRi until relapse or death. Event-free survival (EFS) was calculated from start of induction therapy until induction failure, relapse after CR/CRi or death, whichever occurred first. We performed multivariable Cox regression analysis to evaluate the effects of different variables on OS. Likewise, multi-variable logistic regression models were computed to assess the influence of sex, age, EF, WBC, LDH, blast count, AML type, and genetic risk according to the ELN2010 classification on early death events. *p* values < 0.05 were considered to indicate significant differences. Calculations and plots were performed using the statistical environment R (version 4.2.0).

## Results

Between 2009 and 2020, a total of 31 patients were treated with TAA induction at our department. Patient characteristics are summarized in Table 1. Median (range) age of all patients was 63 (38 –77) years. The most common concomitant cardiac disease was coronary artery disease, which was found in 15 (48%) of these patients, followed by cardiomyopathy in 6 patients (19%) and chronic heart failure, which occurred in 4 patients (13%). The remaining 6 patients suffered from various concomitant cardiac diseases such as atrial fibrillation or severe aortic valve stenosis. If patients had more than one heart condition, they were classified according to the most severe or clinically leading condition. Median (range) EF was 48% (30–67%). Reviewing the medical records of all patients and identified 9 patients with additional noncardiac comorbidities: three patients with type II diabetes, two patients with chronic kidney disease, three patients with a history of stroke or cerebral haemorrhage, and one patient with rheumatoid arthritis. After the first cycle of induction therapy, 13 patients (42%) in this cohort had no residual blasts (< 5%) on a day 15 bone marrow evaluation. Eight (26%) patients did not receive a bone marrow aspirate on day 15, either due to early death or poor general performance status. A second cycle of induction therapy was given in 5 (16.1%) cases. Following one or two induction cycles, 16 patients (52%) achieved CR/CRi.

**Table 1:** Patient characteristics

	TAA induction
No. of patients	31
Age, years, median (range)	63 (38–77)
Sex, *n* (%)	
Female	9 (29)
Male	22 (71.0)
WBC, /nl, median (range)	6.84 (0.5–217)
WBC > 20/nl, n (%)	10 (32)
Hb, g/dl, median (range)	9.1 (5.1–13.9)
LDH, U/l, median (range)	347 (129–3028)
Bone marrow infiltration, median (%)	60
EF, percent, median (range)	48 (30–67)
Cardiac diseases, n (%)	
Coronary heart disease	15 (48)
Cardiomyopathy	6 (19)
Heart failure	4 (13)
Other	6 (19)
ELN 2010 classification, n (%)	
Favourable	3 (10)
Intermediate-I	11 (36)
Intermediate-II	10 (32)
Adverse	7 (23)
AML type,* n* (%)	
De novo	16 (52)
Secondary	13 (42)
Therapy-related	2 (7)

*TAA*, thioguanine, cytarabine, and amsacrine; *WBC*, white blood cell count; *Hb*, hemoglobin; *LDH*, lactate dehydrogenase; *EF*, ejection fraction; *ELN*, European LeukemiaNet

**Fig. 1:**
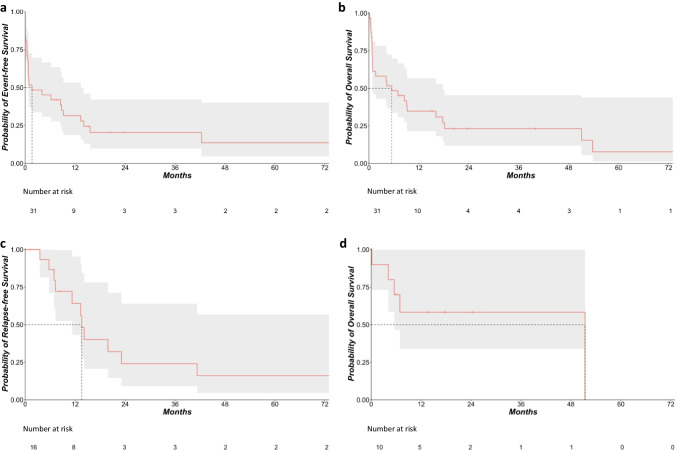
Event-free survival (**a**), overall survival (**b**), and relapse-free survival (**c**) of patients receiving induction therapy with TAA. **d** Overall survival landmarked from the date of transplantation in patients who received a hematopoietic stem cell transplantation

Median EFS was 1.61 months (95% CI 0.72–13.3) (Fig. 1a). Median OS was 5.46 months (95% CI 0.82–17.7) (Fig. 1b). Early mortality within the first 30 days of induction was high, accounting for 12 fatalities (39%). Main cause of death were infectious complications (Table 2). In the 16 patients achieving a CR/CRi, median RFS was 13.6 months (95% CI 11.3–NA) (Fig. 1c).

**Table 2:** Cause of death (30-day mortality)

Type of event	Patients, *n* (%)
Sepsis	8 (67)
Pneumonia	1 (8)
Pulmonary embolism	1 (8)
Ischemic cardiomyopathy	1 (8)
Progress of AML	1 (8)

Of the 31 patients treated with TAA, 10 (32%) patients received an allogeneic HSCT. Median overall survival landmarked from the date of transplantation was 51.4 (95% CI 5.52–NA) months (Fig. 1d).

In multivariable Cox regression analyses, age and genetic risk were significantly associated with OS (HR 1.1 CI 1.02–1.18, *p* = 0.012; HR 0.09 CI 0.01 – 0.85, *p* = 0.035; Table 3). A higher blast count at diagnosis was significantly associated with increased risk of early death in a binary logistic regression analysis (*p* = 0.038).

**Table 3:** Multivariable Cox proportional hazards model for OS

Subgroup	Events/patients	HR	CI	*p*
ELN 2010 classification				
Favourable risk	3/30	0.09	0.01–0.85	**0.04**
Adverse risk	7/30	1.77	0.65–4.84	0.27
AML type				
sAML	13/30	0.63	0.24–1.68	0.36
tAML	2/30	0.35	0.04–2.85	0.33
EF	30/30	0.98	0.94–1.02	0.40
Age	30/30	1.10	1.02–1.18	**0.01**

Hazard ratios (HR) greater or less than 1.0 indicate an increased or decreased risk, respectively, of an event for the higher values of the continuous variables. Significant *p*-values are marked in bold. For ELN 2010 classification, intermediate risk group was used as reference. For AML type, de novo AML was used as reference

## Discussion

In this systematic retrospective single-center analysis, median OS (5.46 months) and 5-year survival (< 10%) following induction therapy with TAA were dismal, mainly because of the high early-death rate related to infectious complications. Our findings are based on a patient cohort treated between 2009 and 2020 and contrast with a former analysis from our department where the TAA induction therapy applied to patients between 1997 and 2003 has been shown to be equally tolerated and efficacious compared to 7 + 3 induction [25]. This discrepancy may result from a smaller sample size of the previous study and the continuous improvement of supportive care strategies since 2008, leading to more patients being referred to intensive treatment despite of comparatively higher comorbidities. Consequently, risk of early death may have increased in our vulnerable cohorts of AML patients over time.

The high early death rate in our analysis exceeds most published rates for intensive AML induction regimens in a cohort of this age [2, 34–36]. Previous studies indicated that amsacrine-based and other common intensive induction regimens are equivalent in AML therapy in terms of toxicity and early death. However, these studies did not necessarily include patients with severe comorbidities [26–31]. Our data suggests unacceptably high treatment-related toxicity of the TAA regimen in AML patients with significant cardiac diseases who were otherwise considered eligible for intensive treatment.

EFS was reduced in our analysis compared with published data, whereas RFS was comparable to published results after induction therapy, underlining the antileukemic efficacy of the TAA regimen [37, 38].

Although our analysis suffers from the limitations of a single-center retrospective study, our results strongly imply that patients with significant concomitant cardiac disease should not be treated with amsacrine-based induction chemotherapy. We believe that whenever application of an anthracycline-based induction regimen is contraindicated, alternative substances should be preferred, e.g., HMA or other targeted therapies. It should be noted that also venetoclax-based therapies may be associated with cardiac complications, even in the absence of pre-existing cardiac disease [39]. However, in terms of response rates, these alternative therapies have been shown to be very effective in AML and do not preclude consolidation with allogeneic HSCT with curative intent [40–43].

## Data Availability

The original data are available by e-mail from the corresponding author on reasonable request and provision of data must be in compliance with the relevant legal regulations.
